# A Case of Placental Site Trophoblastic Tumour That Mimicked Missed Miscarriage

**DOI:** 10.3390/diagnostics16121798

**Published:** 2026-06-11

**Authors:** Joana Paula Artaiz-Pido, Mohd Hafiiz Mohamad Rizan, Kah Teik Chew, Yin Ping Wong, Geok Chin Tan

**Affiliations:** 1 Department of Pathology, Faculty of Medicine, Universiti Kebangsaan Malaysia, Jalan Yaacob Latif, Bandar Tun Razak, Kuala Lumpur 56000, Malaysia; jpsartaiz@gmail.com; 2 Philippine Children’s Medical Center, Quezon Avenue, Cor Senator Miriam P. Defensor-Santiago Ave, Bagong Pagasa, Quezon City 1101, Metro Manila, Philippines; 3 Department of Pathology, Faculty of Medicine and Science Health, University Malaysia Sarawak, Kota Samarahan 94300, Sarawak, Malaysia; mrmhafiiz@unimas.my; 4 Department of Obstetrics and Gynaecology, Faculty of Medicine, Universiti Kebangsaan Malaysia, Jalan Yaacob Latif, Bandar Tun Razak, Kuala Lumpur 56000, Malaysia; drchewkt@gmail.com

**Keywords:** gestational trophoblastic neoplasm, female genital tract, miscarriage, placenta

## Abstract

**Background and Clinical Significance**: Placental site trophoblastic tumour (PSTT) is a malignant tumour of the implantation site intermediate trophoblasts. It has historically been described using terms such as atypical chorioepithelioma, atypical choriocarcinoma, syncytioma, and chorioepitheliosis. It belongs to one of the heterogeneous spectrums of gestational trophoblastic disease. It accounts for about 0.25 to 5% of all gestational trophoblastic neoplasia. The typical clinical presentation is alternating menorrhagia and amenorrhea, mildly elevated β-hCG, and radiological findings of a uterine mass. **Case Presentation**: A 32-year-old woman presented with a history of intermittent menorrhagia and amenorrhea, with a persistent mildly raised β-hCG level. Ultrasonography showed a hypoechoic lesion on the right side of the posterior wall of the uterus. She was diagnosed with a missed miscarriage, and an evacuation of the products of conception was performed. Histologically, the tissue fragments comprised cords and sheets of atypical intermediate trophoblast cells, with characteristic features of myometrial smooth muscle infiltration, vascular invasion, and vascular wall replaced by the neoplastic cells. Immunohistochemically, these cells are positive for β-hCG and GATA3, while negative for P63. **Conclusions**: PSTT is a rare form of gestational trophoblastic neoplasia. Early recognition of PSTT is essential because its clinical presentation may mimic benign pregnancy-related conditions, and diagnosis relies heavily on histopathological and immunohistochemical evaluation.

## 1. Introduction

Gestational trophoblastic disease (GTD) encompasses a spectrum of diseases ranging from non-neoplastic lesions to hydatidiform moles and malignant gestational trophoblastic neoplasia (GTN), which can be further classified into invasive mole, choriocarcinoma, placental site trophoblastic tumour (PSTT), and epithelioid trophoblastic tumour [1,2,3]. PSTT is a rare subtype of GTD that accounts for about 0.25 to 5% of all GTN with an estimated incidence of 1 in 100,000 pregnancies [4]. Given its rarity, it poses a diagnostic dilemma among practicing pathologists. Moreover, the histopathologic features overlap between different types of GTD, like exaggerated placental site, placental site nodule, and epithelioid trophoblastic tumour, and even non-trophoblastic tumours such as squamous cell carcinoma and poorly differentiated carcinoma. We describe a case of a PSTT presented as intermittent menorrhagia and amenorrhea that mimicked a missed miscarriage and discuss the clinicopathological differences between the different types of GTN. This case highlights the importance of considering PSTT in patients with persistent low-level β-hCG elevation and atypical uterine lesions, particularly when clinical findings mimic retained products of conception or missed miscarriage.

## 2. Case Report

### 2.1. Clinical Presentation

A 32-year-old woman, Gravida 2, Para 1, presented to the district clinic with a history of intermittent menorrhagia lasting for about 1 year. The menorrhagia was heavy, requiring up to 3 pads per day and lasting for 7 days, followed by cessation of bleeding for 4 days. It was associated with the passing of a palm-sized blood clot. She did not have a fever, foul-smelling discharge, or abdominal pain. Initially, she was given tranexamic acid. However, the bleeding persisted. Subsequently, she tested positive for a urine pregnancy test. A speculum examination showed an unremarkable cervix, and the Pap smear was normal. Human papillomavirus (HPV) testing was negative for both high-risk and low-risk HPVs. Transvaginal ultrasonography demonstrated no evidence of an intrauterine gestational sac. Notably, the serum beta human chorionic gonadotrophin (β-hCG) level was 354 mIU/mL at 5 weeks 6 days of amenorrhea (POA), and it subsequently was raised to 428 mIU/mL at 6 weeks 4 days POA. She was referred to our hospital for further management. Her past medical and surgical history was unremarkable. Her past obstetric history was unremarkable. Her previous pregnancy was uneventful, and she gave birth to a full-term, healthy baby girl via spontaneous vaginal delivery 9 months prior to the current presentation.

Ultrasonography in our hospital showed an anteverted uterus with a uterine wall thickness of 7.54 cm. No adnexal mass was noted. There was a hypoechoic lesion on the right side at the posterior wall of the uterus, measuring 2.5 × 1.98 cm. At 7 weeks and 4 days POA, she was diagnosed with a missed miscarriage and underwent evacuation of products of conception. There was about 10 mL of evacuated tissue, which was sent for histopathological examination. Subsequent β-hCG monitoring showed it was persistently elevated despite the evacuation. A computed tomography scan (CT scan) was performed post-evacuation, which revealed a heterogeneous enhancing mass within the uterus (Figure 1).

### 2.2. Histopathological Examination

The sample comprised multiple fragments of brownish tissue, measuring 40 mm in aggregate diameter. No gestational sac or vesicle was identified. Microscopically, the tissue fragments are composed of decidual tissue and myometrial tissue infiltrated by intermediate trophoblastic cells arranged in cords and small sheets. These cells demonstrate large, polygonal nuclei, with abundant eosinophilic cytoplasm and irregular cellular borders. Mitoses are observed. Focally, there are up to 3 mitoses per 10 high-power fields. Many multinucleated cells are identified. Areas of necrosis are also observed. No chorionic villi are identified. Vascular invasion is evident, characterised by tumour cells replacing the vascular walls of myometrial vessels (Figure 2).

### 2.3. Immunohistochemical Study

The neoplastic cells are diffusely and strongly positive for GATA3 and show patchy positivity toward β-hCG antibody. They are negative toward the placental-like alkaline phosphatase (PLAP) and P63. Ki67 immunohistochemistry revealed a proliferative index of about 25% (Figure 3).

### 2.4. Management and Follow-Up

After 6 months, she remained ambulatory, with a good appetite and had stable vitals. She had completed the 12th cycle of chemotherapy with the EMA-CO regimen (etoposide, methotrexate, actinomycin D, cyclophosphamide, and vincristine). The most recent serum β-hCG level had returned to normal range, while the radiology imaging demonstrated a reduction in tumour size, with no evidence of regional nodal metastasis.

## 3. Discussion

GTD is a heterogeneous group of disorders that have many overlapping clinicopathological features, making it a diagnostic challenge for many practicing pathologists. PSTT is rare, accounting for <1% of gestational trophoblastic disease. In the majority of cases, about 70% present as irregular bleeding, with or without preceding amenorrhoea. In addition, there is a mild degree of raised β-hCG that could easily mimic a missed miscarriage [5]. This presentation was similarly observed in our case. A study by Betel et al. using ultrasonography found that GTD cases were more likely associated with a larger mass, thin endometrium, myometrial-based mass, and vascular lakes, compared to retained products of conception [6].

Table 1 summarises the clinicopathological features of exaggerated placental site, placental site nodule, PSTT, epithelioid trophoblastic tumour, and choriocarcinoma. In this case, the histological differential diagnoses were exaggerated placental site, PSTT, epithelioid trophoblastic tumour, and choriocarcinoma. With the given clinical presentation of menorrhagia and amenorrhea, mildly elevated β-hCG, and radiological findings of a uterine lesion, our preliminary diagnoses were PSTT, epithelioid trophoblastic tumour, and choriocarcinoma.

The serum β-hCG level in choriocarcinoma is typically markedly elevated compared to PSTT and epithelioid trophoblastic tumour, which are usually mildly elevated [7]. In our case, the serum β-hCG was mildly and persistently elevated. Histologically, PSTT has an infiltrative growth pattern comprised of aggregates of large, polyhedral to round, predominantly mononucleated cells, with scattered multinucleated cells. Notable features are that the neoplastic cells infiltrate the myometrium and vascular wall, and these cells may replace the vascular wall of myometrial vessels [8]. They usually have a low mitotic count [9]. These histological features are observed in our case. Feltmate et al. (2001) found that a high mitotic index is an adverse prognostic indicator for recurrence [10].

The important antibodies for immunohistochemical study to consider are human placental lactogen (hPL), P63, and Ki67 proliferative index markers. hPL, also known as human chorionic somatotropin hormone, is the primary secretory product of the syncytiotrophoblast of the placenta. Studies have shown that the function of hPL includes regulation of the maternal secretion of insulin and raising the glucose level in the foetus. hPL is typically expressed in PSTT and choriocarcinoma, but it is usually weak and focal in epithelioid trophoblastic tumours [11]. On the other hand, P63 is expressed in epithelioid trophoblastic tumour but is negative in PSTT. P63 is a transcription factor, a member of the p53 gene family, and a regulator of epidermal keratinocyte proliferation [12]. The percentage of Ki67 staining is moderate (<30%) in PSTT and epithelioid trophoblastic tumours compared to choriocarcinoma, which is typically >90%. The Ki67 proliferative index in our case is about 25%. (See Table 1). Öz Atalay et al. (2023) [8] described the results of P63 in 9 cases of PSTT and 4 cases of epithelioid trophoblastic tumour, in which all epithelioid trophoblastic tumours were positive with p63, and all PSTTs were negative. GATA3 is 1 of 6 members of the GATA family of transcription factors. It is involved in the luminal differentiation of breast epithelium, the development of urothelium, and trophoblastic differentiation. We also performed GATA-3 to exclude non-trophoblastic carcinoma [13].

PSTT is the second trophoblastic neoplasm that was recognised in the late 1970s. It has historically been described using terms such as atypical chorioepithelioma, atypical choriocarcinoma, syncytioma, and chorioepitheliosis [9]. It is a malignant neoplasm of the implantation site intermediate trophoblast. Two-thirds of the cases follow a full-term pregnancy, with a median latency of 12–18 months (ranging from a few months to 20 years) [14]. The usual age of presentation among patients ranges from 20 to 63 years, with a mean age of 31 years. The most common clinical presentation is vaginal bleeding, uterine enlargement, and mild to moderate elevation of serum β-hCG [7].

In early-stage cases, a simple hysterectomy is usually curative for PSTT. Our patient opted for chemotherapy without a hysterectomy to preserve fertility. PSTT is usually considered to be chemo-resistant. However, there were studies demonstrating complete remission after treated by combined curettage and chemotherapy. A study of 6 cases of PSTT treated by chemotherapy without hysterectomy. After a follow-up for 10 about 47 months, all achieved complete remission and had restored normal menstruation [15]. Numnum et al. (2006) [16] reported a 29-year-old female with a strong desire for fertility who declined surgery. Similar to our case, she was initially thought to have a complete abortion. She declined surgery and was treated with chemotherapy alone who eventually responded well, and her β-hCG returned to normal [16]. Notably, recurrence and metastasis are seen in about 25% of cases. Other treatment modalities include systemic therapy (chemotherapy, targeted therapy, and/or immunotherapy). PD-1/PD-L1 checkpoint inhibitor, like Pembrolizumab, a humanised monoclonal antibody, has been regarded as a potential immunotherapy treatment option in cases of chemotherapy-resistant, metastatic, recurrent, or fertility-preserving situations [17].

PSTT tends to spread through lymph nodes, especially the paraaortic nodes; hence, a lymph node biopsy may be recommended. It generally has a good prognosis, especially in the early stages, where survival rates can reach up to 90–100% after hysterectomy. However, the outcome is poorer in advanced stages and in cases of recurrence or chemotherapy resistance [18,19,20]. In a study of 51 cases diagnosed as GTN treated by chemotherapy, they found pregnancies conceived >12 months after treatment had a better outcome and fewer pregnancy terminations compared to those conceived earlier [21].

Taken together, PSTT can present with highly variable and misleading clinical and imaging features, often resembling more common gynaecological or early pregnancy conditions, which underscores the importance of clinicoradiopathologic correlation.

## 4. Conclusions

In conclusion, PSTT should be in the differential diagnosis of GTD in patients with a myometrial lesion who have a history of intermittent menorrhagia with amenorrhea and a mildly persistent elevated level of serum β-hCG. Both obstetricians and pathologists should be aware of the possibility of PSTT in a case presented with a missed miscarriage. A careful histopathologic examination combined with immunohistochemistry support is crucial in the workup of a suspected case of PSTT. As the prognosis is good in early disease, early diagnosis and timely treatment are key to better clinical outcomes.

## Figures and Tables

**Figure 1:**
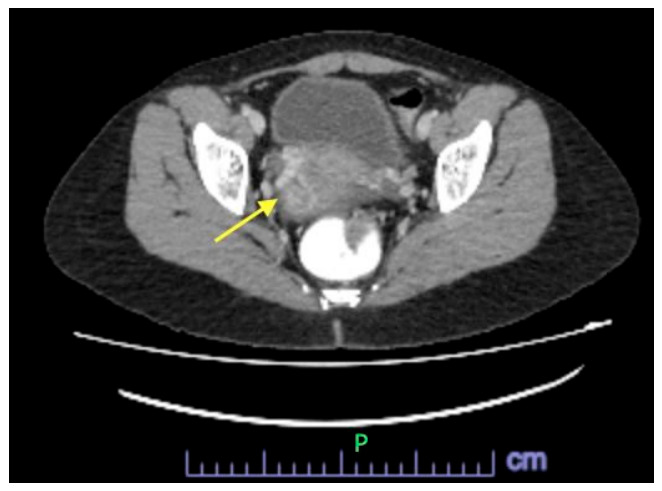
CT scan: There is a heterogeneously enhancing mass within the uterus, measuring 5.4 × 7.1 × 5.4 cm (AP × W × CC) (arrow) with surrounding prominent uterine veins. No clear fat plane seen between the uterine mass and the rectum posteriorly and the urinary bladder anteriorly. P represents posterior in an X-ray picture.

**Figure 2:**
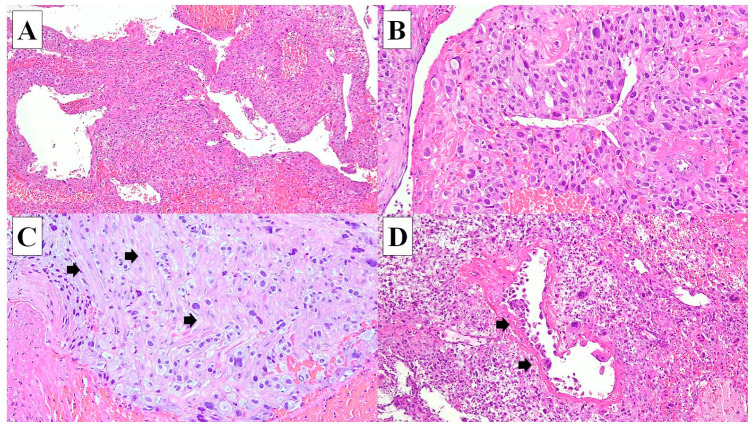
(**A**) The tumour composed of sheets of neoplastic trophoblast cells (×40, H&E). (**B**) The neoplastic cells demonstrate moderate to marked nuclear atypia with pleomorphic and hyperchromatic nuclei and abundant eosinophilic cytoplasm (×200, H&E). (**C**) The myometrial smooth muscle is infiltrated by the neoplastic cells (small arrow showing smooth muscle) (×200, H&E). (**D**) Vascular invasion is identified (small arrows) (×200, H&E).

**Figure 3:**
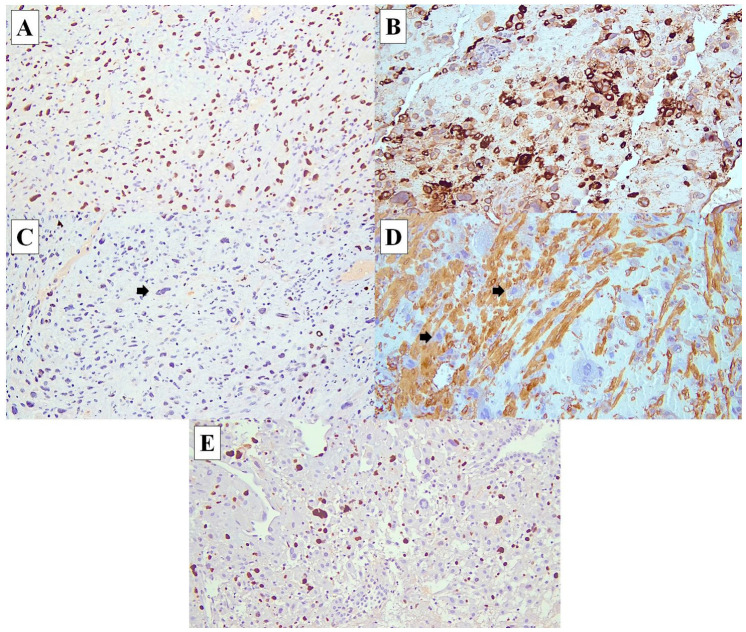
Immunohistochemistry study: The neoplastic cells are positive towards GATA3 ((**A**), ×100) and β-hCG ((**B**), ×100), and they are negative toward P63 ((**C**), ×100). (**D**) The myometrial smooth muscle is highlighted by smooth muscle actin (SMA) with neoplastic cells in between the muscle fibres (×200). (**E**) Ki67 proliferative index is about 20–30% (×100).

**Table 1:** Comparison of the clinicopathological features between the different types of gestational trophoblastic disease, namely exaggerated placental site, placental site nodule, placental site trophoblastic tumour, epithelioid trophoblastic tumour, and choriocarcinoma.

	Exaggerated Placental Site (EPS)	Placental Site Nodule (PSN)	Placental Site Trophoblastic Tumour (PSTT)	Epithelioid Trophoblastic Tumour (ETT)	Choriocarcinoma
**Behaviour**	Non-neoplastic	Non-neoplastic	Neoplastic	Neoplastic	Neoplastic, highly aggressive
**Cell of origin**	Intermediate trophoblast	Chorionic-type intermediate trophoblast	Intermediate trophoblast	Chorionic-type intermediate trophoblast	Villous trophoblast
**Clinical presentation**	Incidental, postpartum, or after abortion	Incidental, asymptomatic	Abnormal uterine bleeding, amenorrhea	Vaginal bleeding	Vaginal bleeding, metastasis (lung, brain)
**β-hCG level**	Normal	Normal	Low or mildly elevated	Mild to moderately elevated	Markedly elevated
**Growth pattern**	Diffuse at the implantation site (no mass)	Small, well-circumscribed nodule	Infiltrative mass in myometrium	Nodular, well-circumscribed with focal invasion	Highly infiltrative & haemorrhagic
**Histological features**	Diffuse infiltration of endometrium and myometrium by intermediate trophoblasts, maybe multinucleated cells.	Well-circumscribed trophoblastic cells in a haphazard pattern, singly, in clusters and cords, surrounded by a thin rim of chronic inflammatory cells and occasionally decidualized stroma. Occasional multinucleated cells.	Confluent sheets of large, polyhedral to round, predominantly mononucleated intermediate trophoblastic cells with scattered multinucleated cells are common. Vascular invasion often present. Tumour cells replace the wall of myometrial vessels.	Nodular, well-circumscribed, expansile growth of medium-sized tumour cells with moderate atypia, arranged in nests, cords, or large sheets. May have a focal infiltrative border. Occasional multinucleated cells. Decidualised stroma cells at the periphery. May have eosinophilic hyaline-like material.	Sheets of syncytiotrophoblast, cytotrophoblast, and intermediate trophoblast (mononuclear and multinucleated cells) with marked nuclear atypia. No chorionic villi. Infiltrative with necrosis and haemorrhage.
**Cellular atypia**	Absent	Generally absent. Occasional large irregular nuclei.	Moderate to marked	Moderate	Marked
**Mitotic activity**	Absent/rare	Absent/rare	Variable, usually low–moderate 2–4 mitoses/10HPF	Highly variable	High
**Necrosis/haemorrhage**	Absent	Absent	Present, minimal & haemorrhage	Present, variable	Present, extensive with haemorrhage
**Ki-67**	Very low (<2%)	Very low (<5%)	Moderate (8–20%)	Moderate to high (10–25%)	Very high (>90%)
**hPL**	Positive	Positive (focal)	Strong diffuse positive	Negative or focal	Positive (focal)
**β-hCG**	Negative/minimal	Negative	Focal	Focal	Strong diffuse
**p63**	Negative	Variable	Negative	Positive (diffuse)	Negative
**Mel-CAM (CD146)**	Positive	Positive	Positive	Negative or rare	Variable
**Inhibin**	Positive	Positive	Positive	Variable	Positive
**SALL4**	Negative	Negative	Negative	Negative	Positive

## Data Availability

No new data were created or analyzed in this study. Data sharing is not applicable to this article.

## Abbreviations

The following abbreviations are used in this manuscript:

Β-hCG	Beta-human Chorionic Gonadotropin
GTD	Gestational trophoblastic disease
GTN	Gestational trophoblastic neoplasm
PLAP	Placental-like alkaline phosphatase
PSTT	Placental site trophoblastic tumour

## References

[1] Shih I.M., Baergen R.N., Cheung A.N.Y., Hui P., Mao T.L., Kaur B. (2020). Gestational trophoblastic disease. WHO Classification of Tumours: Female Genital Tumours.

[2] Chia W.K., Chia P.Y., Abdul Aziz N.H., Shuib S., Mustangin M., Cheah Y.K., Khong T.Y., Wong Y.P., Tan G.C. (2023). Diagnostic utility of TSSC3 and RB1 immunohistochemistry in hydatidiform mole. Int. J. Mol. Sci..

[3] Wong Y.P., Chia W.K., Selimin A., Chia P.Y., Mustangin M., Shuib S., Khong T.Y., Tan G.C. (2021). Diagnostic utility of p57 immunohistochemistry and DNA ploidy analysis by fluorescence in situ hybridisation in hydatidiform moles. Malays. J. Pathol..

[4] Lee H.J., Shin W., Jang Y.J., Choi C.H., Lee J.W., Bae D.S., Kim B.G. (2018). Clinical characteristics and outcomes of placental site trophoblastic tumor: Experience of a single institution in Korea. Obstet. Gynecol. Sci..

[5] Hassadia A., Gillespie A., Tidy J., Everard R.G.N.J., Wells M., Coleman R., Hancock B. (2005). Placental site trophoblastic tumour: Clinical features and management. Gynecol. Oncol..

[6] Betel C., Atri M., Arenson A.M., Khalifa M., Osborne R., Tomlinson G. (2006). Sonographic diagnosis of gestational trophoblastic disease and comparison with retained products of conception. J. Ultrasound Med..

[7] Shih I.M., Kurman R.J. (2002). Molecular basis of gestational trophoblastic diseases. Curr. Mol. Med..

[8] Öz Atalay F., Gündoğdu F., Taşar Kapaklı G.E., Güneş A.C., Güler Y.G., Usubütün A. (2023). Gestational trophoblastic neoplasia of intermediate trophoblasts: Epithelioid trophoblastic tumor and placental site trophoblastic tumor, a study of morphologic, immunohistochemical, and next generation sequencing. Turk. J. Obstet. Gynecol..

[9] Baergen R., Burton G., Kaplan C. (2023). Benirschke’s Pathology of the Human Placenta.

[10] Feltmate C.M., Genest D.R., Wise L., Bernstein M.R., Goldstein D.P., Berkowitz R.S. (2001). Placental site trophoblastic tumor: A 17-year experience at the New England Trophoblastic Disease Center. Gynecol. Oncol..

[11] Alyusuf R.S., Wazir J.F., Brahmi U.P., Fakhro A.R.E., Toorani Z.A., Rezk Y. (2023). The pattern of expression of human placental lactogen across normal, lactational, and malignant mammary epithelium. Cureus.

[12] Yang A., Kaghad M., Wang Y., Gillett E., Fleming M.D., Dötsch V., Andrews N.C., Caput D., McKeon F. (1998). p63, a p53 homolog at 3q27–29, encodes multiple products with transactivating, death-inducing, and dominant-negative activities. Mol. Cell.

[13] Banet N., Gown A.M., Shih I.M., Kay Li Q., Roden R.B., Nucci M.R., Cheng L., Przybycin C.G., Nasseri-Nik N., Wu L.S. (2015). GATA-3 expression in trophoblastic tissues: An immunohistochemical study of 445 cases, including diagnostic utility. Am. J. Surg. Pathol..

[14] Baergen R.N., Rutgers J.L., Young R.H., Osann K., Scully R.E. (2006). Placental site trophoblastic tumor: A study of 55 cases and review of the literature emphasizing factors of prognostic significance. Gynecol. Oncol..

[15] Shen X., Xiang Y., Guo L., Feng F., Wan X., Xiao Y., Ren T. (2012). Fertility-preserving treatment in young patients with placental site trophoblastic tumors. Int. J. Gynecol. Cancer.

[16] Numnum T.M., Kilgore L.C., Conner M.G., Straughn J.M. (2006). Fertility sparing therapy in a patient with placental site trophoblastic tumor: A case report. Gynecol. Oncol..

[17] Mangili G., Sabetta G., Cioffi R., Rabaiotti E., Candotti G., Pella F., Candiani M., Bergamini A. (2022). Current Evidence on Immunotherapy for Gestational Trophoblastic Neoplasia (GTN). Cancers.

[18] Hui P. (2019). Gestational trophoblastic tumors: A timely review of diagnostic pathology. Arch. Pathol. Lab. Med..

[19] Kurman R.J., Ellenson L.H., Ronnett B.M. (2019). Blaustein’s Pathology of the Female Genital Tract.

[20] Feng X., Wei Z., Zhang S., Du Y., Zhao H. (2019). A review on the pathogenesis and clinical management of placental site trophoblastic tumors. Front. Oncol..

[21] McCullough L., Niznanska Z., Danihel L., Sufliarsky J., Kubickova M., Gergely L., Vargova M., Korbel M. (2026). Reproductive outcomes after treatment of gestational trophoblastic neoplasia in Slovakia. Bratisl. Med. J..

